# Identification of carotenoids from the extremely halophilic archaeon *Haloarcula japonica*

**DOI:** 10.3389/fmicb.2014.00100

**Published:** 2014-03-17

**Authors:** Rie Yatsunami, Ai Ando, Ying Yang, Shinichi Takaichi, Masahiro Kohno, Yuriko Matsumura, Hiroshi Ikeda, Toshiaki Fukui, Kaoru Nakasone, Nobuyuki Fujita, Mitsuo Sekine, Tomonori Takashina, Satoshi Nakamura

**Affiliations:** ^1^Department of Bioengineering, Tokyo Institute of TechnologyYokohama, Japan; ^2^Department of Biology, Nippon Medical SchoolKawasaki, Japan; ^3^Department of Biotechnology and Chemistry, Kinki UniversityHiroshima, Japan; ^4^Biotechnology Field, National Institute of Technology and EvaluationTokyo, Japan; ^5^Department of Applied Bioscience, Toyo UniversityGunma, Japan

**Keywords:** extremely halophilic archaeon, *Haloarcula japonica*, C_50_ carotenoid, bacterioruberin, antioxidant capacity

## Abstract

The carotenoids produced by extremely halophilic archaeon *Haloarcula japonica* were extracted and identified by their chemical, chromatographic, and spectroscopic characteristics (UV-Vis and mass spectrometry). The composition (mol%) was 68.1% bacterioruberin, 22.5% monoanhydrobacterioruberin, 9.3% bisanhydrobacterioruberin, <0.1% isopentenyldehydrorhodopin, and trace amounts of lycopene and phytoene. The *in vitro* scavenging capacity of a carotenoid, bacterioruberin, extracted from *Haloarcula japonica* cells against 1,1-diphenyl-2-picrylhydrazyl (DPPH) radicals was evaluated. The antioxidant capacity of bacterioruberin was much higher than that of β -carotene.

## INTRODUCTION

Carotenoids are yellow to red pigments, which originate from the terpenoid biosynthetic pathway. They are synthesized by plants, algae, some fungi, bacteria, and archaea. They are involved in photosynthesis as accessory pigments, and function as antioxidants, light protection pigments, and membrane stabilizers. Their antioxidant properties are closely related to their chemical structure, including aspects such as the number of conjugated double bonds (CDB), the type of structural end-group, and oxygen-containing substituents (Albrecht et al., 2000). Carotenoids are efficient scavengers of reactive nitrogen species, reactive oxygen species (ROS), especially singlet oxygen species, and non-biological radicals (Burton, 1989; Di Mascio et al., 1989; Miller et al., 1996; Chisté et al., 2011).

Extremely halophilic *archaea* generating red-colored colonies produce phytoene, lycopene, β -carotene, acyclic C_50_ bacterioruberin (BR), and its precursors, such as isopentenyldehydrorhodopin (IDR), bisanhydrobacterioruberin (BABR), and monoanhydrobacterioruberin (MABR) (Goodwin, 1980). A halophilic archaeon, *Halobacterium salinarum*, grows chemoorganotrophically in the dark. In the light, they can utilize light energy, even though they still depend on organic nutrients as a carbon source. The molecule responsible for their light utilization is bacteriorhodopsin, which functions as a proton pump to generate ATP through *cis-trans *isomerization of the chromophore retinal, an end product of carotenoid biosynthesis. In the early steps of the carotenoid and retinal biosynthetic pathways, two geranylgeranyl pyrophosphate (GGPP) molecules are condensed to form a C_40_ carotenoid, phytoene, which undergoes a series of desaturation reactions to form the red carotenoid lycopene (Kushwaha et al., 1976). For retinal synthesis, lycopene is cyclized to β -carotene, and then cleaved to a C_20_ retinal cofactor (Peck et al., 2002). Alternatively, lycopene may be used as a precursor for BR, which is a C_50_-xanthophyll functioning to increase membrane rigidity and provide protection against UV light (Lazrak et al., 1998; Shahmohammadi et al., 1998).

*Haloarcula japonica*, the extremely halophilic archaeon, has flat red cells that are predominantly triangular in shape (Takashina et al., 1990; Otozai et al., 1991), suggesting this organism might produce carotenoids. This organism, which requires 2.6–4.3 M NaCl for growth, has a large amount of glycoprotein (CSG) on its cell surface (Nakamura et al., 1992; Nishiyama et al., 1992; Horikoshi et al., 1993). By using flash-induced fluorescence spectroscopic analysis, a bacteriorhodopsin-like retinal protein was identified on the cell envelope vesicles of *Haloarcula japonica* (Yatsunami et al., 1997). These results suggest that* Haloarcula japonica* has both carotenoids and retinal biosynthetic pathways. Recently, the draft genome sequence of *Haloarcula japonica* has been determined (Nakamura et al., 2011). However, the carotenoid composition and both carotenoids and retinal biosynthetic pathways of *Haloarcula japonica* have not been identified yet.

Here, we present the carotenoid composition of *Haloarcula japonica* and evaluate the antioxidant potential of an extracted carotenoid using the 1,1-diphenyl-2-picrylhydrazyl (DPPH) method and compare its activity with that of β-car-otene.

## MATERIALS AND METHODS

### STRAIN AND CULTIVATION CONDITIONS

Extremely halophilic archaeon *Haloarcula japonica* strain TR-1 (JCM 7785^T^) was pre-cultured at 37°C in the dark with a complex medium as described previously (Das Sarma and Fleoschmann, 1995). 4 ml of pre-inoculum was transferred to a 2 L Erlenmeyer flask containing 400 mL of the liquid medium and cultured to a stationary phase for 10 days under the same conditions. The cells were harvested by centrifugation at 4,400 × *g* for 20 min, washed with a basal salt solution [20% (w/v) NaCl and 4%(w/v) MgSO_4_·7H_2_0], and stored at -80°C until used.

### EXTRACTION OF CAROTENOIDS

The extraction of the carotenoids was performed under dim light as follows. A frozen cell pellet was thawed, and 10 times volume of acetone/methanol (7:3, v/v) was added. The suspension was sonicated with a sonic oscillator (VP-5S, Taitec, Koshigaya, Japan) for several seconds and centrifuged. The supernatant was collected and evaporated. The carotenoids were dissolved in a small volume of *n*-hexane/acetone and loaded onto a DEAE-Toyopearl 650 M (Tosoh, Tokyo, Japan) column to remove the polar lipids (Takaichi and Ishidzu, 1992). The non-adsorption fraction including the carotenoids was recovered and evaporated. The carotenoids were also dissolved in a small volume of *n*-hexane/acetone and loaded on a column of silica gel 60 (Merck, Darmstadt, Germany). Separation was achieved by binary graduation elution using an initial composition of 90% *n*-hexane and 10% acetone, which was decreased stepwise to 50% *n*-hexane and 50% acetone. All fractions were recovered, evaporated, and further analyzed by HPLC with a μBondapack C_18_ column (8 × 100 mm, RCM type, Waters, Milford, MA, USA), as described previously (Takaichi and Ishidzu, 1992). The elution was performed with 100% methanol at 1.8 ml min^-^^1^. The absorption spectra were recorded with a photodiode-array detector (250–580 nm, 1.3-nm intervals, MCPD-3600, Otsuka Electronics, Osaka, Japan) attached to the High-performance liquid chromatography (HPLC) apparatus as described previously (Takaichi and Shimada, 1992). The peaks of lycopene and phytoene were collected again, further separated by HPLC with Novapack C_18_ column (8 × 100 mm, RCM type, Waters) and eluted with a mixture of acetonitrile, methanol, and tetrahydrofuran (58:35:7, v/v) (2.0 ml min^-^^1^) as described previously (Takaichi, 2000). The lycopene and phytoene were identified by a combination of the HPLC retention times and the absorption spectra. Other carotenoids, including IDR, BABR, MABR, and BR were detected with absorbance at 490 nm. To identify the each elution peak, the relative molecular masses of the purified carotenoids were measured by field-desorption mass spectrometry using a double-focusing gas chromatograph/mass spectrometer equipped with a field-desorption apparatus (M-2500, Hitachi, Tokyo, Japan) according to the method of Takaichi (1993). The 500 MHz ^1^H NMR spectrum of the BR was recorded in CDCl_3_ at 25°C on a Varian VXR-500S spectrometer (Varian Medical Systems, Palo Alto, CA, USA).

### QUANTIFICATION OF CAROTENOIDS

For the quantification of carotenoids, ethyl β -apo-8′-carotenoate (Wako Pure Chemical, Osaka, Japan) was used as an internal standard. 10 μl of 0.5 mM ethyl β-apo-8′-carotenoate in ethanol was added to the samples upon extraction. The suspension was disrupted by sonication with a Ultrasonic disruptor UD-201 (Tomy Seiko, Tokyo, Japan) for several seconds and centrifuged. The supernatant was collected and evaporated. The carotenoids were dissolved in a small volume of *n*-hexane/acetone, analyzed by HPLC with a μBondapack C_18_ column (3.9 × 300 mm, 125 Å, 10 μm, Waters). The HPLC system consisted of a SCL-10A chromatograph fitted with a photodiode-array detector (SPD-M20A, Shimadzu, Kyoto, Japan) and controlled with an LC solution (Shimadzu). The carotenoids were eluted with methanol/water (9:1) for the first 10 min and then with 100% methanol (1.5 ml min^-^^1^). Detection was performed at 490 nm, and the online spectra were acquired in the 190–800 nm wavelength range with 1.2 nm resolution. Each carotenoid was identified by the retention time on the HPLC and the absorption spectrum in the eluent by a photodiode-array detector. The absorption coefficients of BR and its derivatives at 490 nm were assumed to be 167 mM^-1^ cm^-^^1^ (Kelly et al., 1970). That of the ethyl β -apo-8′-carotenoate at 445 nm was assumed to be 100 mM^-^^1^ cm^-^^1^, which is the same as that of the β -apo-8′-carotenoic acid (Isler et al., 1959).

### DPPH RADICAL SCAVENGING ASSAY

DPPH radical scavenging activity was measured using an ESR spectrometer (JES-FA-100, JEOL, Tokyo, Japan). The stable free radical, DPPH was dissolved in acetone (200 μM). β -Carotene, a standard antioxidant, was used as a positive control. The BR and β -carotene were diluted in 100 μl of acetone, yielding concentrations of 0–200 and 0–800 μM, respectively. The 100 μl DPPH solution and the 100 μl carotenoid solution were mixed, and the DPPH radical was measured after 60 s. The spin adduct was detected by ESR spectrometer exactly 2 min later. The ESR measurement conditions were as follows: field sweep, 330.500–340.500 mT; field modulation frequency, 100 kHz; field modulation width, 0.25 mT; sweep time, 2 min; time constant, 0.1 s; microwave frequency, 9.427 GHz; and microwave power, 4 mW. All the scavenging activities in the present study were calculated using the following equation, in which H and H_0_ were the peak areas of the radical signals with and without a sample, respectively:

Radical scavenging activity (%) = [1-(H/H_0_)] × 100.

## RESULTS

### CHARACTERIZATION OF CAROTENOID PROFILES OF *Haloarcula* japonica

**FIGURE 1:**
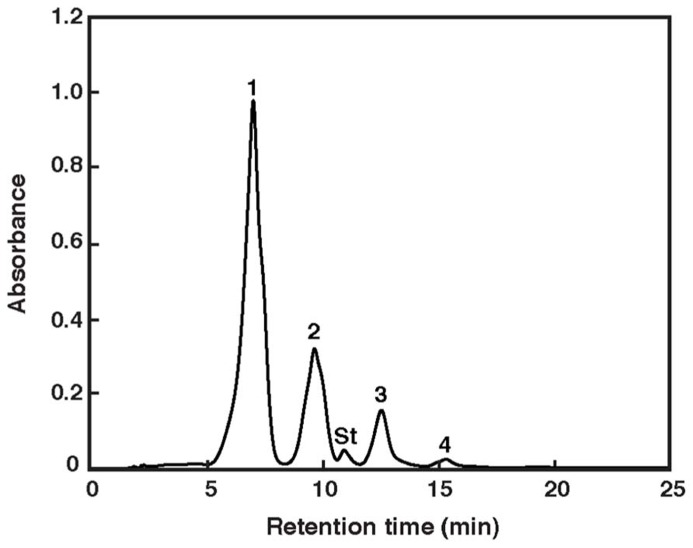
**Elution profile of pigments extracted from *Haloarcula japonica.*** The HPLC system consisted of a reversed-phase μBondapack C_18_ column. Absorbance at 490 nm is shown. Peak identification is revealed in **Table 1**. St: internal standard of ethyl β-apo-8′-carotenoate.

**FIGURE 2:**
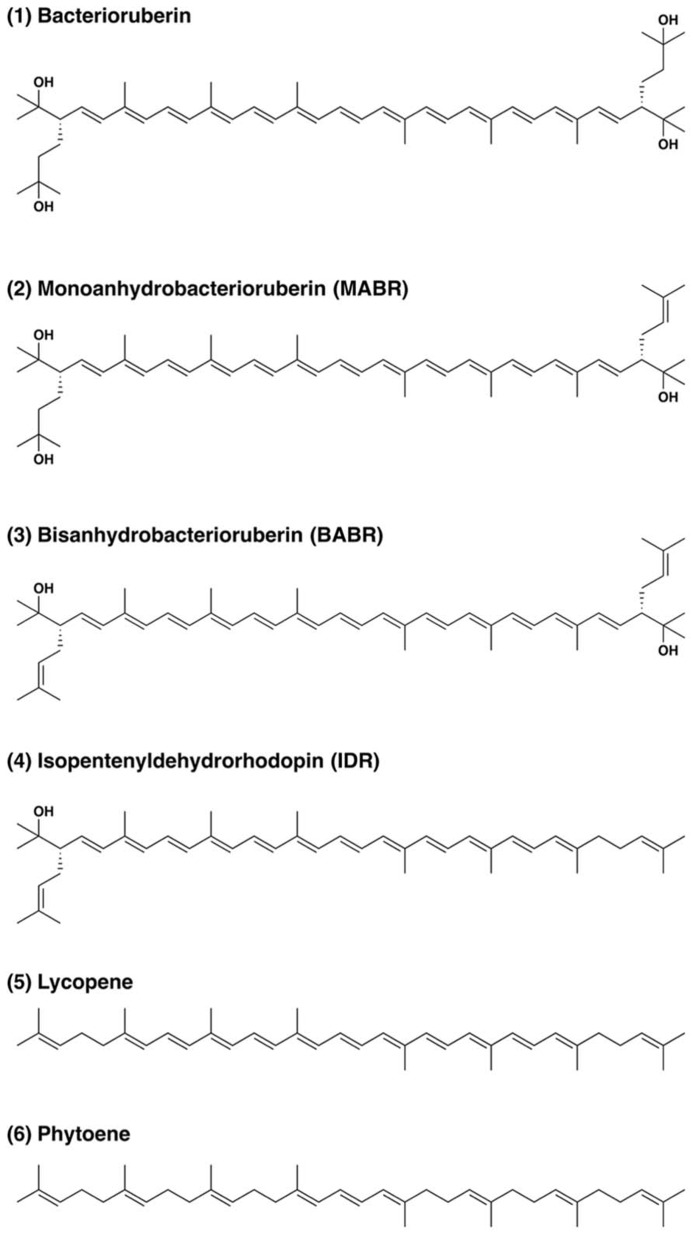
**Structure of carotenoids produced by *Haloarcula japonica***.

The carotenoids occurring in *Haloarcula japonica* were extracted and identified based on their chemical, chromatographic, and spectroscopic characteristics (UV-Vis and mass spectrometry). **Figure 1** shows an elution profile on HPLC system that corresponds to the carotenoids obtained from *Haloarcula japonica*. **Table 1** summarizes the identification for each chromatographic peak, and **Figure 2** shows the corresponding chemical structures. Peak 1, which was the major carotenoid, was assigned as all-*trans*-BR. The UV-Vis spectrum, mass spectrum, CD spectrum (data not shown), and NMR spectra (data not shown) were compatible with those of BR from *Haloferax volcanii* (Rønnekleiv and Liaaen-Jensen, 1995). Peaks 2 and 3 were similarly identified as MABR and BABR, respectively. Peak 4 was a minor C_45_-carotenoid, IDR. In addition to these carotenoids, two minor C_40_ carotenoids were also eluted at 19.4 min. They were identified as phytoene and lycopene using another HPLC system (date not shown). These carotenoids were also found in *Halobacterium salinarum* (Kelly et al., 1970).

**Table 1:** Characteristics of carotenoids produced by *Haloarcula japonica*.

Peak^a^	Carotenoid	Retention time (min)	λ_max_ (nm) in methanol	[M]^+^(*m/z*)
1	BR	7.0	387, 466, 488, 525	741.0	
2	MABR	9.6	369, 385, 465, 492, 524	722.8	
3	BABR	12.5	370, 386, 460, 492, 524	704.5	
4	IDR	15.3	375, 455, 480, 511	620.7	
–	Lycopene	19.4	296, 363, 445, 472, 501^b^	not done
–	Phytoene	19.4	277, 288, 298^b^	not done

a Peak numbers are based on ***Figure 1*** using μBondapack C_18_ column.

b λ_max_ in acetonitrile/MeOH/THF (58:35:7) using Novapack C_18_ column.

### QUANTITATIVE ANALYSIS OF CAROTENOIDS

The total carotenoid content was 335 μg g^-^^1^ of dry mass, although the contents in *Halobacterium salinarum *and* Halococcus morrhuae* were 89 and 45 μg g^-^^1^, respectively (Mandelli et al., 2012). This contents were about four and seven times compared to that of *Halobacterium salinarum *and *Halococcus morrhuae*, respectively. BR was the major pigment, accounting for up to 68.1% of the total carotenoids (mol%). Therefore, it was the main one responsible for the red color of this organism. The BR content in *Haloarcula japonica* was similar to those in other halophilic archaea (Mandelli et al., 2012). Other major pigments were MABR (22.5%) and BABR (9.3%), and IDR was found at a lower level (<0.1%). These results suggest that BR is produced as a final product in *Haloarcula japonica* and is synthesized from other C_50_ carotenoids, such as IDR, BABR, and MABR as well as other halophilic archaea.

### ANTIOXIDANT CAPACITY

**FIGURE 3:**
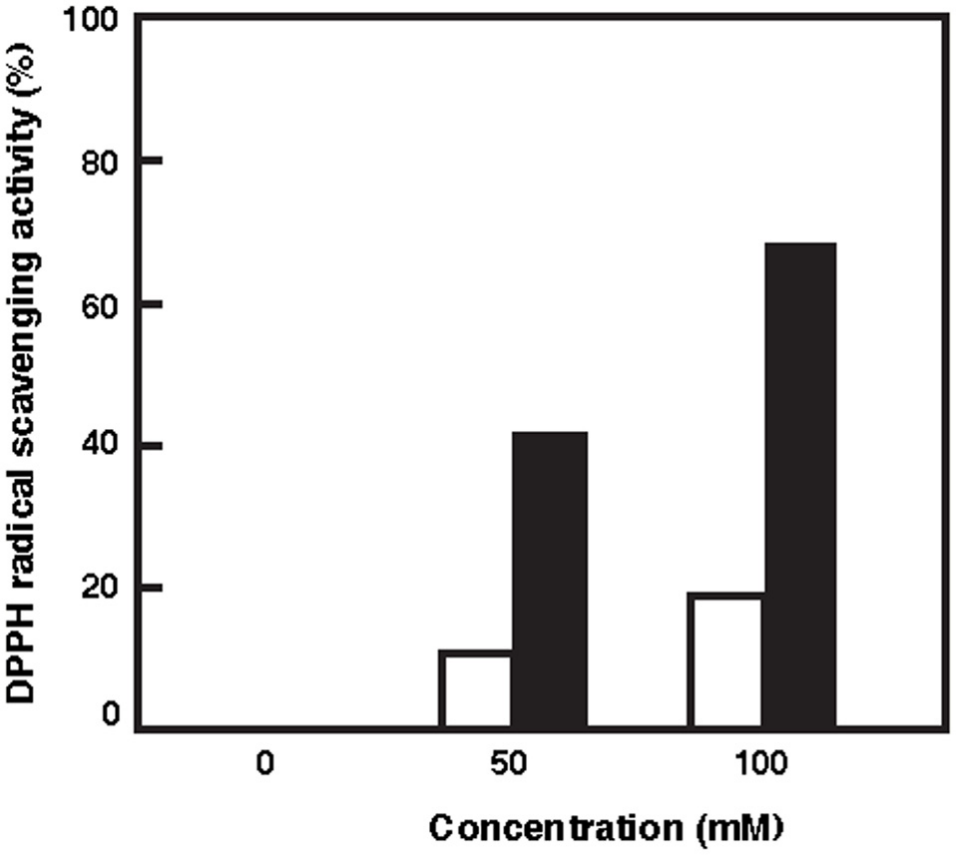
**Radical scavenging activities of BR and β-carotene against DPPH radicals.** White bars, β-carotene; Black bars, BR.

An ROS formed under photo-oxidation stress can react with macromolecules like lipids and proteins and cause a cellular damage. Antioxidants are substances that have the ability to reduce ROS and prevent macromolecules from oxidation (Klein et al., 2012). DPPH method was carried out to evaluate the antioxidant capacity of the carotenoid extracted. ESR spin trapping provides a sensitive, direct, and accurate means of monitoring reactive species (Guo et al., 1999). DPPH is a stable free radical donor, which is widely used to test the free radical scavenging effect of natural antioxidants. DPPH method involves the scavenging of a performed stable radical by an electron transfer mechanism from the carotenoid to the radical, generating a carotenoid radical cation (Huang et al., 2005). The scavenging capacity of the reactive species was dependent on the carotenoid concentration. **Figure 3** shows that the scavenging capacity of BR was much higher than that of β-carotene. The oxidant capacity seems to relate to both the length of the CDB and the presence of the function group. The antioxidant capacity of the carotenoids increases with increased extension and maximum overlap of the CDB molecular orbitals (Albrecht et al., 2000; Tian et al., 2007). BR molecule contains 13 CDB, which are much more than the nine CDB of the β-carotene. Therefore, the BR would be a good DPPH radical scavenger.

## DISCUSSION

In the present study, we extracted the carotenoid pigments occurring in *Haloarcula japonica* and identified them. This is the first report concerned with the carotenoids produced by the extremely halophilic archaeon *Haloarcula japonica*. The production of carotenoids from *Haloarcula japonica* is very attractive. Previous data suggested that the C_50_-carotenoids found in halophilic archaea may be incorporated in membranes due to their length, and that their two polar end-groups may facilitate the adjustment to such membranes; moreover, it has been reported that baterioruberin reinforces the lipid membrane of *Halobacterium* spp.(Ourisson and Nakatani, 1989).

In addition, the carotenoids synthesized by these microorganisms have a function to protect their cells against the lethal actions of ionizing radiation, UV radiation, and hydrogen peroxide (Shahmohammadi et al., 1998). Saito et al. (1997) have extracted BR from *Rubrobacter radiotoleranse. *They studied the OH scavenging effect using a system of thymine degradation and compared with that of β-carotene. These results have shown that the OH radical scavenging ability of BR was much higher than that of β-carotene. In this work, the scavenging capacity of BR extracted from *Haloarcula japonica* toward DPPH free radicals was measured. BR exhibited higher DPPH free radical scavenging than β-carotene. The present result was consistent with the previous study.

Since the carotenoids produced by halophilic archaea can play both roles of membrane stabilization and protection against oxidizing agents, these compounds are essential for the survival of such microorganisms. In order to clarify the function of BR *in vivo*, further studies using of BR-deficient *Haloarcula japonica* mutant were needed.

## Conflict of Interest Statement

The authors declare that the research was conducted in the absence of any commercial or financial relationships that could be construed as a potential conflict of interest.
